# Baihe Zhimu formula attenuates the efficacy of tamoxifen against breast cancer in mice through modulation of CYP450 enzymes

**DOI:** 10.1186/s12906-019-2651-0

**Published:** 2019-09-04

**Authors:** Hailong Li, Chunyu Wu, Yu Liu, Shuo Zhang, Xiufei Gao

**Affiliations:** 1 Zhejiang University of Technology, No. 18, Chaowang Road, Zhejiang, 310014 Hangzhou China; 2 Department of Breast Surgery (Integrated Traditional and Western Medicine), Longhua Hospital, Shanghai University of Traditional Chinese Medicine, No. 725 South Wanping Road, Shanghai, 200032 China; 3 Department of Breast Surgery, The First Affiliated Hospital of Zhejiang University of Traditional Chinese Medicine, No. 54, Youdian Road, Zhejiang, 310006 Hangzhou China

**Keywords:** Baihe Zhimu formula, Tamoxifen, Drug-drug interaction, CYP450, Breast cancer, Depressive disorder

## Abstract

**Background:**

Major depression is an important complication in patients with breast cancer, but is an underrecognized and undertreated condition in this population. The Baihe Zhimu Tang (BZ formula) is a traditional Chinese formula consisting of *Lilium brownii var. viridulum* Baker (*L. brownii*) and *Anemarrhena asphodeloides* (*A. asphodeloides*) Bunge that is used for the treatment of depression. However, the interaction between tamoxifen and BZ formula is frequently overlooked by traditional and alternative medical doctors. In the present study, the influence of BZ formula on the effectiveness of tamoxifen in breast cancer in mice and the effects of tamoxifen on the antidepressant effect of BZ formula and its major components mangiferin and timosaponin BII in mice were investigated.

**Methods:**

Identification of the major components of BZ formula was performed using fast HPLC-tandem mass spectrometry (HPLC-MS/MS). The main flavonoids and saponins in *A. asphodeloides* were determined by HPLC-UV and HPLC-ELSD, separately. The antidepressant efficacy of BZ formula was evaluated using a mouse tail-suspension test. The effects of BZ formula on the antineoplastic activity of tamoxifen were performed in a mouse xenograft model of human breast cancer MCF-7 cells. P450 activity was determined using microsomal incubations by HPLC-MS/MS. Measurement of serum concentrations of tamoxifen and its metabolites was used by HPLC-MS/MS.

**Results:**

BZ formula attenuated the effectiveness of tamoxifen treatment of breast cancer and reduced the concentrations of endoxifen and 4-OH-tamoxifen in tumor-bearing mice. Of two of the major components of BZ formula, the antidepressant effect of mangiferin, but not timosaponin BII, was significantly inhibited by tamoxifen in mice. BZ formula and its component mangiferin also significantly inhibited CYP450 enzyme activity in rat liver microsomes.

**Conclusion:**

BZ formula attenuated the effectiveness of tamoxifen in treatment of breast cancer in mice by influencing CYP450 enzymes. The present study laid a foundation for the treatment of patients with breast cancer and depression by BZ formula or other Chinese herbal formulas containing *A. asphodeloides*.

## Background

Tamoxifen, a selective modulator of the estrogen receptor, has been the focus of research and a treatment method for breast cancer for over 50 years and remains recommended as a standard therapy for estrogen receptor positive (ER^+^) breast cancer by the Clinical Pharmacogenetics Implementation Consortium [1]. Administration of tamoxifen reduces the annual recurrence rate by almost half and cancer mortality by one-third in women with ER^+^ breast cancer [2]. Extensive primary and secondary metabolism of tamoxifen, including conversion to N-demethylation and 4-hydroxylation, occurs in the liver by cytochrome P450 (CYP450) enzymes. One major conversion is demethylation of tamoxifen to N-desmethyltamoxifen, which is primarily facilitated by CYP3A4 [3], followed by oxidation to 4-hydroxy-N-desmethyltamoxifen, i.e., endoxifen, by CYP2D6 [4]. Tamoxifen drug-drug interactions are controversial, but careful evaluation of previously published clinical evidence suggests CYP2D6 inhibitors and inducers may dampen the efficacy of tamoxifen [5].

Major depression is an important complication of breast cancer and has a prevalence in this population of up to 9.3% [6]. Within the first year after diagnosis, patients with breast cancer are at high risk for depression, particularly if they are premenopausal, are less than 65 years old, have a history of depression, or received chemotherapy [7]. Unfortunately, major depression is a condition that is underrecognized and undertreated among patients with breast cancer [6]. In addition, only 27% of these patients received antidepressant drugs or visited a mental health professional [6]. Paroxetine is a generally well tolerated and effective drug for treating major depression and is mainly metabolized by CYP450 enzymes in the liver [8].Therefore, administering paroxetine to women with breast cancer concurrently taking tamoxifen should be avoided due to drug interactions involving CYP450 enzymes [9].

Accumulating evidence indicates there are potential benefits from using complementary and alternative medicine to treat cancers and major depressive disorder [10, 11]. Survivors of breast cancer have strong tendencies to utilize these types of treatments, particularly herbal remedies and natural product supplements. Up to 60% of cancer survivors have taken traditional Chinese medicines despite these medicines having questionable efficacy and safety [12]. The herbal pair Baihe Zhimu (Baihe Zhimu Tang, BZ) consists of *Lilium brownii var. viridulum* Baker (*L. brownii var. viridulum*, Baihe in Chinese) and *Anemarrhena asphodeloides* Bunge (*A. asphodeloides*, Zhimu in Chinese) and is a traditional Chinese formula used for the treatment of depression. BZ formula was first described by Zhang Zhongjing, a famous Chinese physician who lived from 150 to 219 A.D., in “Jingui Yaolue” for the treatment of “Lily syndrome”. BZ formula has been used to mitigate the symptoms of depression, anxiety, and stress-related illness in patients in China [13]. Animal experiments confirmed this medication is effective for the treatment of depression [14]. The main components of BZ formula are flavonoids, saponins, and phenolic glycosides [15, 16], such as neomangiferin, mangiferin, isomangiferin, timosaponin BII, timosaponin BIII, timosaponin AIII, regaloside A, regaloside B, regaloside D, and regaloside E. Recently, Idania Rodeiro et al. found mangiferin inhibits P450 enzymes and UDP-glucuronosyltransferases in human hepatocytes [17]. However, the drug-drug interaction between tamoxifen and BZ formula has not been addressed in previous studies. Therefore, the drug-drug interaction between BZ formula and tamoxifen requires urgent attention. In the present study, the influence of BZ formula on the effectiveness of tamoxifen in breast cancer in mice was investigated and a possible primary mechanism was identified.

## Methods

### Chemicals

Fetal bovine serum and RPMI 1640 medium were obtained from Gibco (Grand Island, NE, USA). Modified Lowry protein assay kits were procured from Pierce (Rockford, IL, USA). Tamoxifen, 4-hydroxytamoxifen, endoxifen, phenacetin, paracetamol, dextromethorphan, and dextrorphan were purchased from Sigma (St. Louis, MO, USA). Midazolam and 1-hydroxymidazolam were obtained from Gentest (Woburn, MA, USA). HPLC-grade formic acid and acetonitrile were obtained from Fisher (Dayton, OH, USA). Deionized water was purified with a Milli-Q system (Millipore, Bedford, MA, USA). All other chemicals, unless otherwise indicated, were procured from Sigma-Aldrich (St. Louis, MO, USA).

### Plant material and extraction

*A. asphodeloides* rhizomes and *L. brownii var. viridulum* bulbs were purchased from Shanghai Cambridge Traditional Chinese Medicine Co., Ltd. (Shanghai, China). The identities of the plants were made by source identification, morphological examination and microscopic identification in comparison to herbarium specimens by a senior Traditional Chinese medicine pharmacist Professor Caihua Sun in our hospital. Dried samples of *A. asphodeloides* and *L. brownii var. viridulum* were mixed at a 1:3 ratio, placed in a flask containing 10-fold (100 g/L) distilled water, and soaked for 2 h. This mixture was then boiled for 2 h and filtered and the filter residue was treated again using the same method. Afterwards, the two decoctions were combined and concentrated to a dry powder. Twenty-two g of dry powder was equivalent to 100 g of the crude herbs. This powder was kept at 4 °C until experimental use. Stock solutions were generated from the powder, where 100 mg/mL solutions were prepared by dissolving powder in DMSO for cell experiments and 200 mg/mL solutions were prepared in saline for mouse experiments.

### Cells and cell culture

Human GFP-labeled MCF-7 and hepatocyte L02 cells were obtained from the Shanghai Institute of Cell Biology, Chinese Academy of Sciences (Shanghai, China). The cells were cultured in RPMI 1640 medium containing 10% fetal bovine serum and 2 μg/mL purinomycin at 37 °C in a humidified atmosphere containing 5% CO_2_. Cancer cells expressing GFP were selected for using 2 μg/mL purinomycin and identified by fluorescence-activated cell sorting. The purinomycin was removed prior to using these cells in vivo to establish the mouse xenograft model.

### Animals

Female nude and male ICR mice (20 ± 2 g) were purchased from Shanghai SLAC Laboratory Animal Co., Ltd. (Shanghai, China) and then housed under specific pathogen-free and temperature-controlled (24 ± 2 °C) conditions with a regular 12-h light/dark cycle. The mice were provided water and food ad libitum throughout the experiments. All experiments were carried out according to the national regulations for animal experimentation and were approved by the Zhejiang Chinese Medical University Institutional Animal Care and Use Committee.

### Identification of the major components of BZ formula

To identify the main components of BZ extract, fast HPLC-tandem mass spectrometry (HPLC-MS/MS) was performed using an Agilent 1200 HPLC system (Agilent Technologies, Wilmington, DE, USA) coupled with an Agilent 6300 series ion trap mass spectrometer (Agilent technology, Palo Alto, CA, USA) as described in previous work [15]. An Apollo C18 chromatographic column (4.6 × 250 mm, 5 μm) was obtained from Grace Technologies (Hangzhou, China). A Dikma EasyGuard Kit C18 guard column (2.1 mm × 12.5 mm, 5 μm) was purchased from Dikma Technologies (Shanghai, China). An Agilent C18 solid-phase extraction column (100 mg, 1 mL) was obtained from Agilent (Agilent technology, Palo Alto, CA, USA). The mobile phase was 0.1% formic acid solution in acetonitrile and the flow rate was 0.5 mL/min. Gradient separation was programmed as shown in Table 1.

**Table 1:** Gradient Elution Conditions

Type	Time (min)	A (%)	B (%)
HPLC-MS/MS		Acetonitrile	0.1% formic acid solution
	0–8	5	95
	8–10	10	90
	10–20	20	80
	20–50	20	80
	50–70	55	45
	70	100	0
HPLC-UV		Acetonitrile	0.3% formic acid solution + 0.05% Triethylamine solution
	0–12	8–13	92–87
	12–27	13	87
	27–40	13–24	87–76
	40–56	24	76
	56–57	24–100	76–0
HPLC-ELSD		Acetonitrile	0.3% formic acid solution + 0.05% Triethylamine solution
	0–15	24	76
	15–20	24–35	76–65
	20–30	35	65
	30–35	35–100	65–0
	35–50	100	0

The main flavonoids in *A. asphodeloides*, such as mangiferin and neomangiferin, were identified by HPLC-DAD using an Agilent 1260 HPLC system (Agilent technology, Palo Alto, CA, USA) as described in previous work [18]. Briefly, the chromatographic separation was performed on a reverse-phase Inertsil ODS-3 column (150 mm × 4.6 mm, 5 μm; GL Sciences, Tokyo, Japan) protected with a guard column (10.0 mm × 4.6 mm, 5 μm; Dikma Technologies, Beijing, China) with a mobile phase of acetonitrile-water containing 0.1% formic acid (16:84, v/v) eluted at 1.0 ml/min. The wavelength was set at 320 nm, and the column temperature was kept at 25 °C. The injection volume was 20 μl.

The main saponins in *A. asphodeloides*, such as timosaponin BII and timosaponin BIII, were identified by HPLC-ELSD using an Agilent 1260 HPLC system (Agilent technology, Palo Alto, CA, USA) as described in previous work [19]. Briefly, the chromatographic separation was used a C18 column in methanol-water containing 0.5% acetic acid (30:70, v/v) eluted at 1.0 ml/min. The injection volume was 20 μl.

### MTT assay

Cell viability was measured using MTT assay kits as described previously [20]. Briefly, MCF-7 tumor cells were incubated with the indicated concentrations of BZ formula or one of its main active representative components, timosaponin BII and mangiferin for 24 h. While in L02 cell viability analysis, cell were incubated indicated the indicated concentrations of BZ formula or one of its main active representative components for 48 h. An equal volume of DSMO was used as the vehicle. The optical densities from triplicate wells for each treatment cohort were converted to percentage of the mean vehicle group.

### Mouse xenograft model and treatments

The mouse xenograft model was established by injecting 2 × 10^6^ cells/mouse GFP-labeled MCF-7 cells subcutaneously. The mice bearing tumors one week post-injection were then divided randomly into five groups according to the size of tumor with 5 mice per group. Each group was administered one of 5 treatments intragastrically once-daily for 8 consecutive weeks: (1) tamoxifen (4 mg/kg), (2) tamoxifen (4 mg/kg) + paroxetine (4 mg/kg), (3) tamoxifen (4 mg/kg) + BZ formula (2 g/kg), (4) tamoxifen (4 mg/kg) + BZ formula (8 g/kg), or (5) an equal volume of vehicle (0.5% CMC-Na). The dissolution of tamoxifen and paroxetine was used 0.5% CMC-Na. The volume of administration (po) was 10 ml/kg. The BZ formula dose was determined by conversion from the equivalent clinical recommended dose of ~ 40 g/day (30–50 g *L. brownii var. viridulum* Baker and 10–15 g *A. asphodeloides*). All experiments were performed in accordance with the national regulations for animal experimentation approved Shanghai University of Traditional Chinese Medicine.

### Fluorescence imaging in vivo

Fluorescence imaging was carried out according to our previous publication [21]. An IVIS Lumina II in vivo imaging system was used to image the mice (Caliper Life Sciences, USA) according to the manufacturer’s recommended procedures.

### Tail-suspension test

The antidepressant efficacy of BZ formula was evaluated using a mouse tail-suspension test [22]. Briefly, mice were administered the indicated drugs intragastrically once daily for 7 consecutive days. The vehicle was 0.5% CMC-Na solution. The volume of administration (po) was 10 ml/kg. The tail-suspension test was performed 1 h at after the final administration of drug. The duration of immobility during the last four minutes of a total of 6 min was recorded using a video camera and scored using SuperTst software (Shanghai Xinruan Information Technology Co. Ltd). The animals were euthanized in CO2-rich atmosphere after the experiment.

### Rat liver microsome preparation

Male SD rats (200 ± 20 g) were obtained from Shanghai SLAC Laboratory Animal Co., Ltd. (Shanghai, China). All animals were anaesthetized by intraperitoneal administration of 40 mg/kg pentobarbital after food deprivation for 15 h. Rat liver microsomes were obtained by centrifugation as previously described from isolated perfused rat livers [23]. All experiments were carried out at 0–4 °C. Rat liver microsomes were immediately placed at − 80 °C. Protein levels were measured using modified Lowry assays.

### P450 enzyme activity assays

P450 activity was determined using microsomal incubations as described previously [24]. HPLC-MS/MS analysis was performed on an Agilent 1100 HPLC system (Agilent Technologies, Wilmington, DE, USA) coupled with a Xevo TQ-XS triple-quadru-pole mass spectrometer (Waters Technologies, Milford, MA, USA). Phenacetin and its metabolite paracetamol were used to determine CYP1A2 activity. Dextromethorphan and its metabolite dextrorphan were used to determine CYP2D6 activity. Midazolam and its metabolite 1-Hydroxymidazolam were used to determine CYP3A4 activity.

### Measurement of serum concentrations of tamoxifen and its metabolites

Tamoxifen, 4-hydroxytamoxifen (4-OH-tamoxifen), and endoxifen were measured according to previous work [25]. HPLC-MS/MS analysis was performed on an Agilent 1260 HPLC system (Agilent Technologies, Wilmington, DE, USA) coupled with an AB SCIEX Triple Quad™ 4500 LC/MS/MS System (AB SCIEX, Concord, Ontario, Canada).

### Statistical analysis

All results are expressed as mean ± standard deviation. One-way analysis of variance (ANOVA) followed by Dunnett’s post hoc analysis was used to identify statistically significant differences between groups.

## Results

### Identification of the main components of BZ formula by HPLC-MS/MS and HPLC

**Fig. 1:**
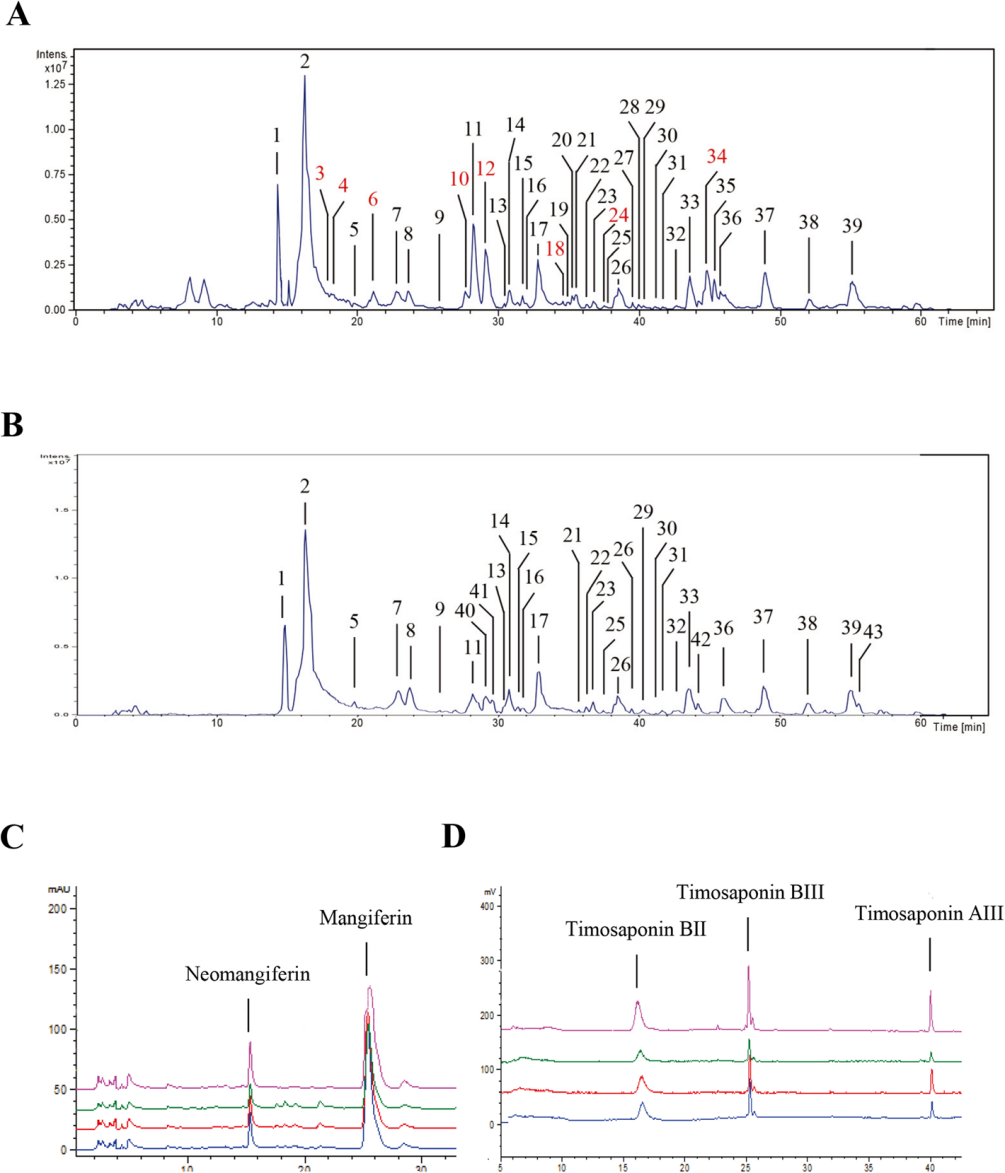
Main components of Baihe Zhimu formula and *Anemarrhena asphodeloides* extract. **a** Thirty-nine compounds were identified in Baihe Zhimu (BZ) formula by HPLC-MS/MS in negative ion mode, as shown in Table 2. **b** Thirty compounds were identified in *A. asphodeloides* by HPLC-MS/MS, as shown in Table 3. The fingerprints of the main components in *A. asphodeloides* were analyzed by **c** HPLC-UV and (**d**) HPLC-ELSD

The main components of the BZ formula extract identified by HPLC-MS/MS were flavonoids, saponins, and phenolic glycosides, including mangiferin, neomangiferin, isomangiferin, timosaponin BII, timosaponin BIII, timosaponin AIII, regaloside A, and regaloside B (Table 2). These results are consistent with previous work [15, 16]. The chemical fingerprint was shown in Fig. 1a. The same method was used to analyze the main components of *A. asphodeloides*. We found that the main flavonoids and saponins in BZ formula, including mangiferin, neomangiferin, isomangiferin, timosaponin BII, timosaponin BIII, and timosaponin AIII, originated from *A. asphodeloides* based on contrast analysis and previous work, as shown in Fig. 1b and Table 3 [15, 16]. Correspondingly, regaloside A, regaloside B, regaloside D, regaloside E, hydroxyl deacylbrownioside, and 26-O-β-D-glucopyranosyl-3β,26-dihydroxy-5-choleslen-16,22-dioxo-3-O-β-L-rhamnopy-ranosyl-(1 → 2)-β-D-glucopyranoside originated from *L. brownii var. viridulum* (Table 2). We further analyzed the main compounds in *A. asphodeloides* using HPLC and found that the neomangiferin, mangiferin, timosaponin B II, timosaponin B III, and timosaponin A III concentrations were 3.0, 16.9, 35.0, 15.5, and 6.9 mg/g, respectively (Fig. 1c, d and Table 4).

**Table 2:** The components of BZ formula identified by HPLC-MS/MS

No.	Identification	RT (min)	Molecular formula
1	Neomangiferin	14.4	C_25_H_28_O_16_
2	Mangiferin	17.4	C_19_H_18_O_11_
3	Regaloside A	17.9	C_18_H_24_O_10_
4	Regaloside D	18.2	C_19_H_26_O_10_
5	Isomangiferin	19.9	C_19_H_18_O_11_
6	Regaloside E	21.2	C_18_H_24_O_10_
7	Mangiferin isomer	22.8	C_19_H_18_O_11_
8	Vitexin	23.7	C_21_H_20_O_10_
9	Timosaponin E1	25.9	C_45_H_76_O_20_
10	Regaloside B	27.8	C_20_H_26_O_11_
11	Timosaponin E	28.2	C_46_H_78_O_19_
12	4-acetyl derivative of regaloside D	29.2	C_20_H_26_O_11_
13	Timosaponin D isomer	30.4	C_45_H_74_O_19_
14	Timosaponin N	30.7	C_45_H_76_O_20_
15	Timosaponin D	31.8	C_45_H_74_O_19_
16	Timosaponin D isomer	32.0	C_45_H_74_O_19_
17	Timosaponin BII	32.9	C_45_H_76_O_19_
18	26-O-β-D-glucopyranosyl-3β,26-dihydroxy-5-choleslen-16,22-dioxo-3-O-β-L-rhamnopy-ranosyl-(1 → 2)- β-D-glucopyranoside or its isomer	34.6	C_45_H_72_O_18_
19	Timosaponin BII isomer	34.9	C_45_H_76_O_19_
20	Timosaponin D	35.3	C_45_H_74_O_19_
21	Timosaponin BII isomer	35.6	C_45_H_76_O_19_
22	Timosaponin BII isomer	36.2	C_45_H_76_O_19_
23	Timosaponin BIII	36.8	C_45_H_74_O_18_
24	26-O-β-D-glucopyranosyl-3β,26-dihydroxy-5-choleslen-16,22-dioxo-3-O-β-L-rhamnopy-ranosyl-(1 → 2)-β-D-glucopyranoside or its isomer	37.5	C_45_H_72_O_18_
25	Timosaponin F (C39)	37.8	C_39_H_64_O_13_
26	Timosaponin BIII isomer	38.6	C_45_H_74_O_18_
27	Norathyriol	39.6	C_13_H_8_O_6_
28	Timosaponin BIII isomer	39.9	C_45_H_74_O_18_
29	Timosaponin AII isomer	40.3	C_39_H_64_O_14_
30	Timosaponin BII isomer	41.2	C_45_H_76_O_19_
31	Anemarrhenasaponin Ia	41.6	C_40_H_68_O_18_
32	Anemarrhenasaponin I	42.6	C_39_H_66_O_14_
33	Timosaponin AII isomer	43.6	C_39_H_64_O_14_
34	Hydroxyl deacylbrownioside	44.7	C_57_H_93_O_29_
35	Timosaponin AII isomer	45.2	C_39_H_64_O_14_
36	Timosaponin AII	45.8	C_39_H_64_O_14_
37	Timosaponin AIV	48.9	C_39_H_64_O_13_
38	Timosaponin AIII isomer	52.0	C_39_H_64_O_13_
39	Timosaponin AIII	55.1	C_39_H_64_O_13_

**Table 3:** The components of *Anemarrhena asphodeloides* identified by HPLC-MS/MS

No.	Identification	RT (min)	Molecular formula
1	Neomangiferin	14.9	C_25_H_28_O_16_
2	Mangiferin	16.4	C_19_H_18_O_11_
5	Isomangiferin	19.9	C_19_H_18_O_11_
7	Mangiferin isomer	22.8	C_19_H_18_O_11_
8	Vitexin	23.7	C_21_H_20_O_10_
9	Timosaponin E1	25.9	C_45_H_76_O_20_
11	Timosaponin E	28.2	C_18_H_24_O_10_
13	Timosaponin D isomer	30.4	C_45_H_74_O_19_
14	Timosaponin N	30.7	C_45_H_76_O_20_
15	Timosaponin D	31.4	C_45_H_74_O_19_
16	Timosaponin D isomer	31.8	C_45_H_74_O_19_
17	Timosaponin BII	32.9	C_45_H_76_O_19_
21	Timosaponin BII isomer	35.8	C_45_H_76_O_19_
22	Timosaponin BII isomer	36.2	C_45_H_76_O_19_
23	Timosaponin BIII	36.8	C_45_H_74_O_18_
25	Timosaponin F (C39)	37.4	C_39_H_64_O_13_
26	Timosaponin BIII isomer	38.6	C_45_H_74_O_18_
29	Timosaponin AII isomer	40.3	C_39_H_64_O_14_
30	Timosaponin BII isomer	41.2	C_45_H_76_O_19_
31	Anemarrhenasaponin Ia	41.6	C_40_H_68_O_18_
32	Anemarrhenasaponin I	42.7	C_39_H_66_O_14_
33	Timosaponin AII isomer	43.6	C_39_H_64_O_14_
36	Timosaponin AII	46.0	C_39_H_64_O_14_
37	Timosaponin AIV	48.9	C_39_H_64_O_13_
38	Timosaponin AIII isomer	52.0	C_39_H_64_O_13_
39	Timosaponin AIII	55.1	C_39_H_64_O_13_
40	Timosaponin E1	29.1	C_45_H_76_O_20_
41	Timosaponin E	29.6	C_18_H_24_O_10_
42	Deglycosyl timosaponin BV	44.2	C_40_H_68_O_14_
43	Timosaponin AIII isomer	55.7	C_39_H_64_O_13_

**Table 4:** The content of the main compounds content in *A. asphodeloides* as measured by HPLC

Names	Content (mg/g)
Neomangiferin	3.0 ± 0.2
Mangiferin	16.9 ± 1.4
Timosaponin BII	35.0 ± 3.2
Timosaponin BIII	15.5 ± 2.8
Timosaponin AIII	6.9 ± 2.3

### BZ formula attenuated the effectiveness of tamoxifen in mice

**Fig. 2:**
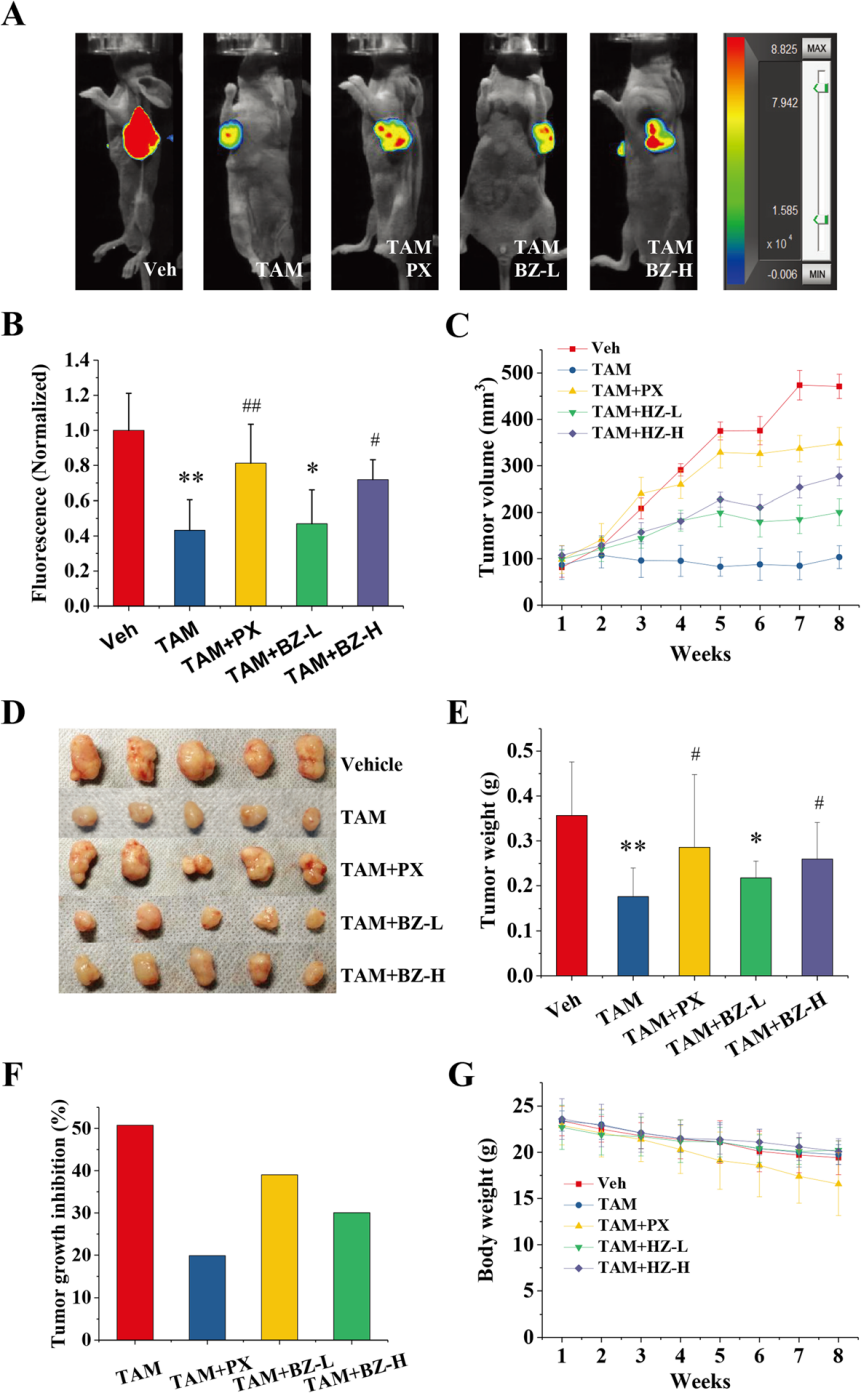
BZ formula attenuated the effectiveness of tamoxifen against breast cancer in mice. Nude mice were injected with estrogen receptor-positive (ER^+^) human breast cancer MCF-7 cells and then treated with 4 mg/kg tamoxifen (TAM) alone or in combination with BZ formula (BZ-L/BZ-H, 2 or 8 g/kg) or paroxetine (PX, 4 mg/kg). The control group was treated with an equal volume of physiological saline. There were five animals per group. **a** Representative bioluminescent images of tumor-bearing mice. **b** Normalized bioluminescent signals for mice from each experimental group. * *p* < 0.05 and ** *p* < 0.01 compared to the vehicle group based on one-way ANOVA. # p < 0.05 and ## p < 0.01 compared to the tamoxifen group based on one-way ANOVA. **c** Xenograft tumor growth as measured by volume during treatment. **d** Comparison of tumor samples excised from vehicle and treatment groups. **e** Final xenograft tumor weight. * p < 0.05 and ** p < 0.01 compared to the vehicle group based on one-way ANOVA. # p < 0.05 and ## p < 0.01 compared to the tamoxifen group based on one-way ANOVA. **f** Comparison of tumor suppressor rate. **g** Changes in body weight of tumor-bearing mice after 8 weeks of treatment with tamoxifen alone or in combination with BZ or paroxetine

After 8 weeks of treatment, cancer cell proliferation was significantly reduced by tamoxifen in tumor-bearing mice (Fig. 2). Normalized fluorescence in vivo, tumor volume, and tumor weight were reduced by ~ 57, 77, and 51%, respectively, compared with the vehicle group (Fig. 2a-e). Inhibition of the tumor growth rate by tamoxifen reached 51% (Fig. 2f). Unfortunately, paroxetine significantly reduced the efficacy of tamoxifen (Fig. 2). There was no significant difference between combination use of tamoxifen and paroxetine and the vehicle groups (Fig. 2d, e). In addition, inhibition of the tumor growth rate by treatment with the combination of tamoxifen and paroxetine was less than 20% (Fig. 2f). In addition, we found a high dose of BZ formula (8 g/kg) significantly reduced the efficacy of tamoxifen (Fig 2). There were no significant differences between the combination of the high dose of BZ formula and tamoxifen and the vehicle group based on normalized fluorescence in vivo and tumor weight (Fig. 2a, b, d, e). The reduction in efficacy by BZ was less pronounced when a low dose (2 g/kg) was administered (Fig 2). These data indicated that the antagonistic effect of BZ formula was a dose-dependent. In terms of bodyweight, no significant differences were observed between any of the groups of tumor-bearing mice (Fig. 2g).

### BZ formula reduced the concentrations of endoxifen and 4-OH-tamoxifen in mice

**Fig. 3:**
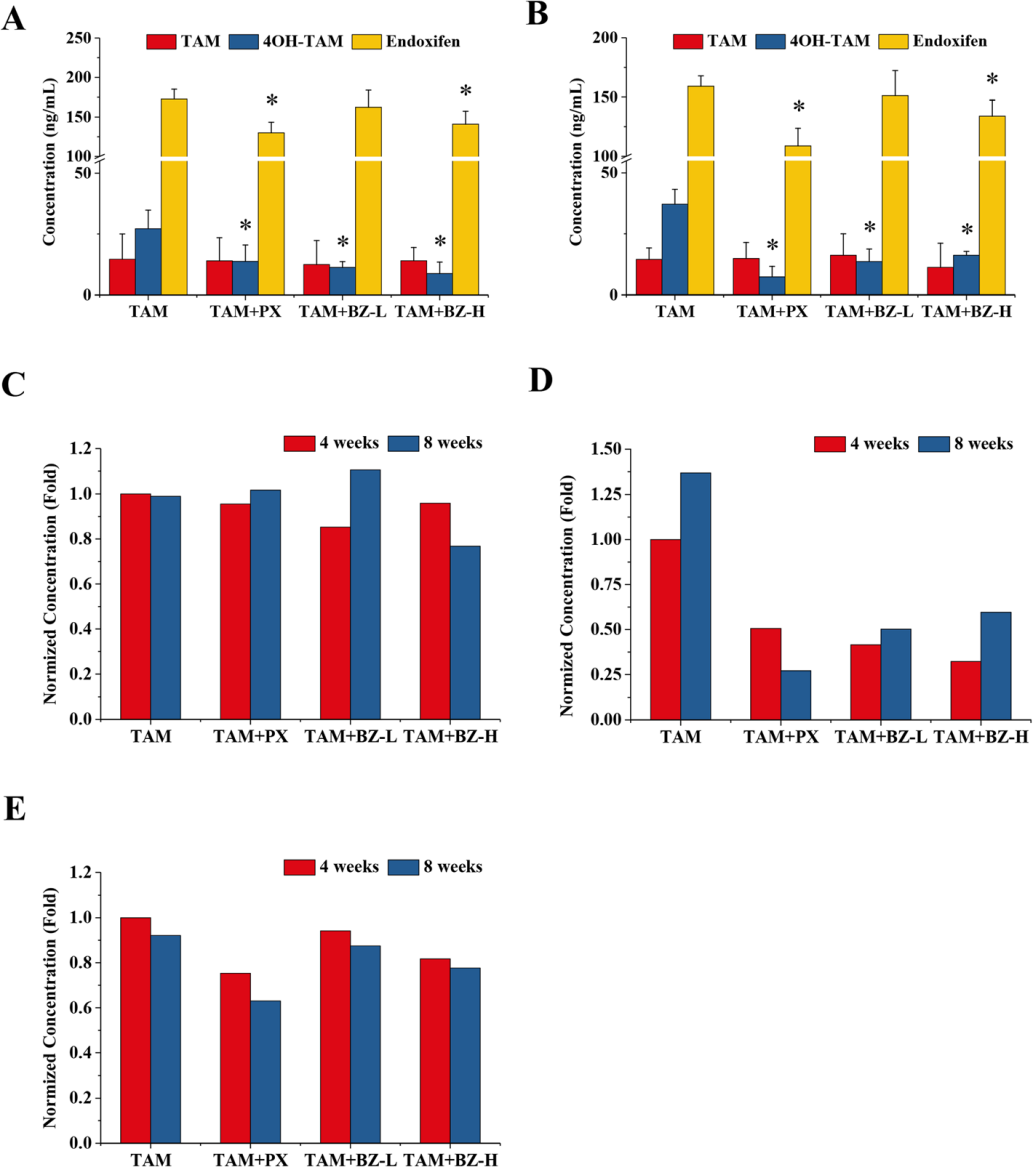
Administration of BZ formula with tamoxifen decreased endoxifen and 4-OH-tamoxifen concentrations in tumor-bearing mice. Nude mice were injected with ER^+^ human breast cancer MCF-7 cells and then treated with 4 mg/kg tamoxifen alone or in combination with BZ formula (2 or 8 g/kg) or paroxetine (4 mg/kg). The control group was treated with an equal volume of physiological saline. There were five animals per group. Blood was collected 1 h after oral administration on the last day of treatment and stored at -80 °C after centrifugation at 12000 rpm for 10 min. The serum tamoxifen and endoxifen concentrations were measured using HPLC-MS/MS. The tamoxifen, endoxifen, and 4-OH-tamoxifen concentrations after **a** 4 and **b** 8 weeks. *p < 0.05 compared to the tamoxifen group based on one-way ANOVA. Normalized concentrations of **c** tamoxifen, **d** 4-OH-taxoxifen, and **e** endoxifen in the tamoxifen group after treatment for 4 and 8 weeks

Blood samples were collected from the experimental mice 1 h prior to treatment at weeks 4 and 8. HPLC-MS/MS analysis revealed that the concentrations of tamoxifen at weeks 4 and 8 were not significantly different between any treatment groups (Fig. 3a, b). Surprisingly, the concentrations of endoxifen and 4-OH-tamoxifen in mice administered a combination of high dose BZ formula and tamoxifen or paroxetine and tamoxifen were significantly lower than in the tamoxifen group (Fig. 3a, b). When normalized to the concentrations of tamoxifen, 4-OH-tamoxifen, and endoxifen in the tamoxifen only group after 4 weeks of treatment, the concentrations of 4-OH-tamoxifen and endoxifen, but not tamoxifen, were discernably lower in the combination groups than the tamoxifen only group (Fig. 3c-e).

### Effects of tamoxifen on the antidepressant effect of BZ formula in mice

**Fig. 4:**
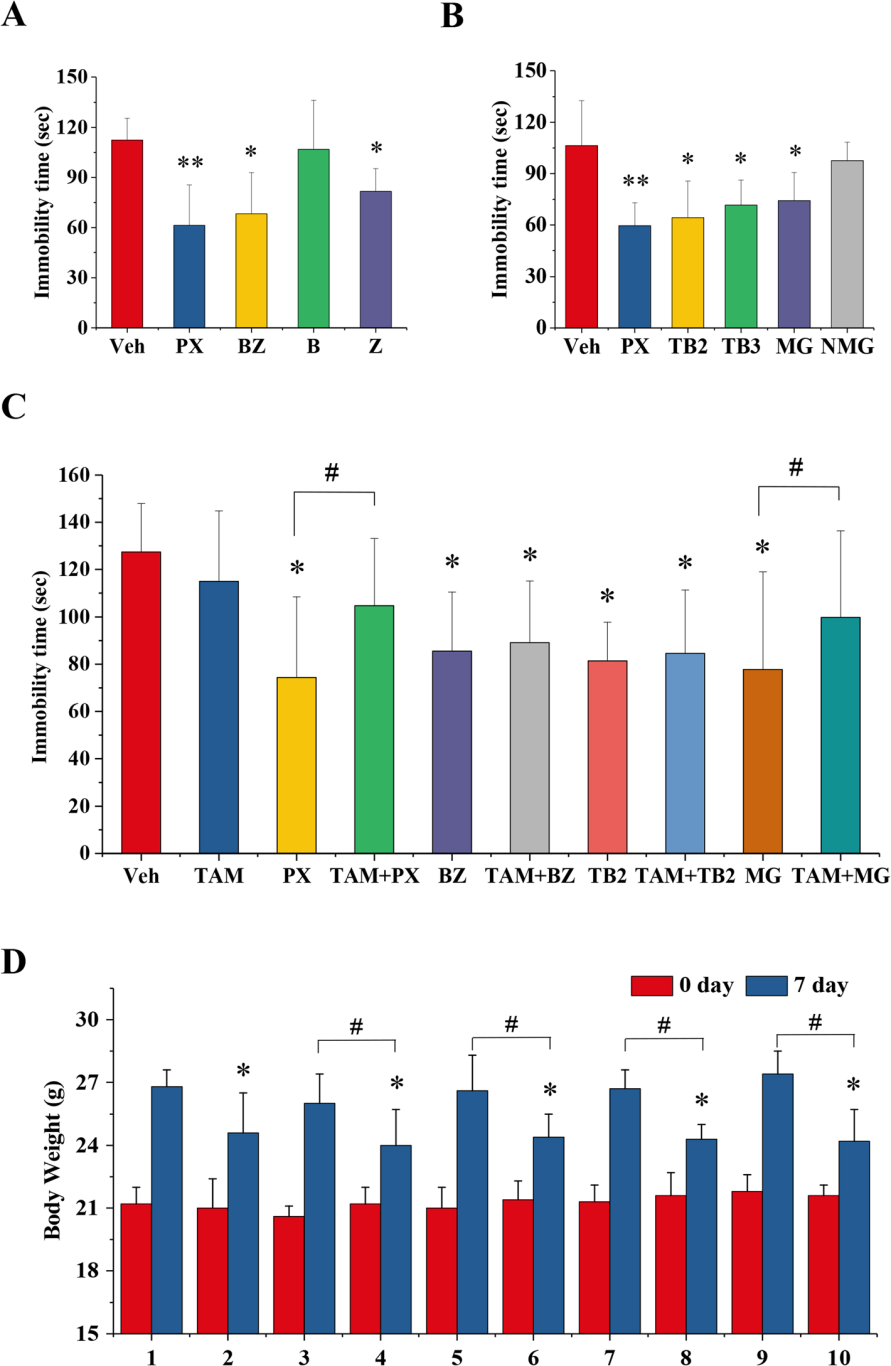
The effects of tamoxifen on the antidepressant efficacy of BZ formula in mice. Antidepressant analysis was performed using the tail-suspension test after treatment for 7 days. There were ten animals per group. **a** The antidepressant effect of BZ formula is derived from the Zhimu rather than the Baihe. ICR mice were treated with paroxetine (4 mg/kg), BZ formula extract (4 g/kg), Baihe extract (B, 4 g/kg), or Zhimu extract (Z, 4 g/kg). The control group was treated with an equal volume of physiological saline. The test was performed 1 h after treatment. The duration of immobility over the last four minutes of a total of 6 min was recorded using a video camera and scored using SuperTst software (Shanghai Xinruan Information Technology Co. Ltd). * p < 0.05 and ** p < 0.01 compared to the vehicle group based on one-way ANOVA. **b** The antidepressant effect of the main components of Zhimu. Mice were treated with paroxetine (4 mg/kg), timosaponin BII (TB2, 20 mg/kg), timosaponin BIII (TB3, 20 mg/kg), mangiferin (MG, 20 mg/kg), or neomangiferin (NMG, 20 mg/kg). The control group was treated with an equal volume of physiological saline. * p < 0.05 compared to the vehicle group based on one-way ANOVA. (C) The effects of tamoxifen on the antidepressant effect of BZ formula in mice. Mice were treated with paroxetine (4 mg/kg), tamoxifen (4 mg/kg), TB2 (20 mg/kg), MG (20 mg/kg), BZ formula extract (4 g/kg) alone or in combination with tamoxifen (4 mg/kg). The control group was treated with an equal volume of physiological saline. * p < 0.05 compared to the vehicle group based on one-way ANOVA. # p < 0.05 compared to their individual group based on one-way ANOVA. **d** Body weight changes in ICR mice in the experiment in **c**. 1: Vehicle; 2: tamoxifen; 3: paroxetine; 4: tamoxifen + paroxetine; 5: BZ formula; 6: tamoxifen + BZ formula; 7: timosaponin BII; 8: tamoxifen + timosaponin BII; 9: mangiferin; 10: tamoxifen + mangiferin. * p < 0.05 compared to the vehicle group based on one-way ANOVA. # p < 0.05 compared to their individual group based on one-way ANOVA

In order to study the potential antidepressant components in BZ formula, mouse tail-suspension tests were performed. As shown in Fig. 4a, BZ formula and its component *A. asphodeloides* Zhimu significantly reduced the duration of immobility. We further explored the potential antidepressant effects of the major BZ formula components timosaponin BII, timosaponin BIII, mangiferin, and neomangiferin. Timosaponin BII, timosaponin BIII, and mangiferin significantly decreased the duration of immobility in mice (Fig. 4b). To study the potential effects of tamoxifen on the antidepressant effects of BZ formula and its main components, we examined the effect of treating mice with a combination of tamoxifen and BZ formula or its main components. We found tamoxifen significantly reduced the antidepressant effect of paroxetine (Fig. 4c). Similar antagonistic effects were also observed when mice were administered both tamoxifen and mangiferin (Fig. 4c). There were no obvious differences between the groups administered BZ formula and tamoxifen, and timosaponin BII and tamoxifen, BZ formula alone, or timosaponin BII alone (Fig. 4c). In addition, tamoxifen significantly reduced the bodyweight gains measured post-treatment in mice compared to the vehicle group (Fig. 4d). There were no differences between most of the individual treatment groups given paroxetine, BZ formula, timosaponin BII, or mangiferin, with the exception of the tamoxifen group (Fig. 4d). In addition, the body weight gain measured post-treatment in mice treated only with tamoxifen was significantly lower than for mice in the vehicle group (Fig. 4d), as well as the combination treatment group compared to the vehicle or individual treatment groups (Figure 4d). However, no significant differences were observed between the groups for the bodyweights of tumor-bearing mice (Fig. 2g). These results suggest tamoxifen may have unpleasant side effects in normal, but not tumor-bearing, mice. One possible reason this occurs may be the loss of weight in tumor-bearing mice masked this difference.

### BZ formula and mangiferin inhibited CYP450 enzyme activity

**Fig. 5:**
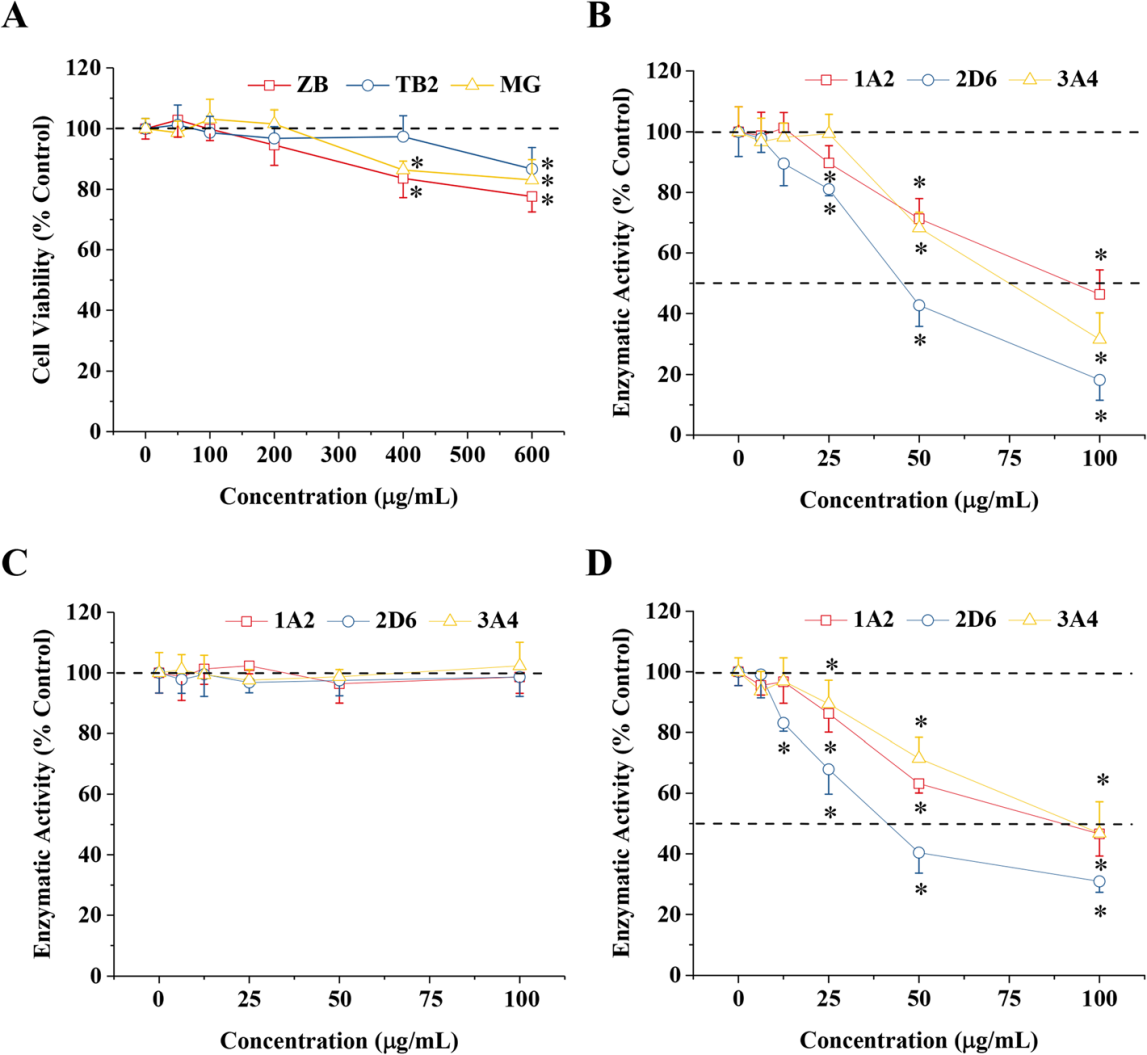
Effects of BZ formula and its main components, timosaponin BII and mangiferin, on P450 activity in rat liver microsomes. **a** Cytotoxicity of BZ formula and its main components on human L02 hepatocytes was measured 48 h after treatment using the MTT test. The effects of **b** BZ formula, **c** timosaponin BII, and **d** mangiferin on P450 activity in rat liver microsomes. After a 1-h incubation, the microsomes were exposed to the indicated concentrations of BZ formula, timosaponin BII, or mangiferin. Results are expressed as the percentage of cell viability compared to the controls (untreated microsomes). Each point represents the mean ± SD of three replicates. * *p* < 0.05 compared to the vehicle group based on one-way ANOVA

In order to avoid the use of cytotoxic concentrations of BZ formula and its main components, timosaponin BII and mangiferin, we carried out MTT viability assays using L02 hepatocytes. No obvious cytotoxicity was observed in the three treatment groups when the treatment concentration was ≤200 μg/mL (Fig. 5a). Therefore, concentrations ≤100 μg/mL were used in the subsequent experiments assessing CYP450 enzyme activity. We found BZ formula and mangiferin significantly inhibited CYP1A2, CYP2D6, and CYP3A4 activity when present at concentrations higher than 25 μg/mL (Fig. 5b, d). However, timosaponin BII did not regulate CYP450 enzyme activity (Fig. 5c). The IC50 values for BZ formula for CYP1A2, CYP2D6, and CYP3A4 activity were 93, 46, and 74 μg/mL and for mangiferin were 91, 41, and 94 μg/mL, respectively (Table 5).

**Table 5:** Effects of BZ formula and its main components timosaponin BII and mangiferin on P450 activity in rat liver microsomes

Names	CYP450s	IC50 (μg/mL)
ZB formula	1A2	93.2 ± 2.1
	2D6	45.6 ± 2.6
	3A4	74.4 ± 6.2
Timosaponin B II	1A2	> 100
	2D6	> 100
	3A4	> 100
Mangiferin	1A2	90.6 ± 5.1
	2D6	41.3 ± 4.3
	3A4	93.8 ± 2.2

## Discussion

Drug-drug interactions play key roles in increasing and decreasing therapeutic benefits, toxicity, and adverse events, especially in targeted chemical drugs [26]. Some patients with ER^+^ breast cancer could benefit from treatment considerations for tamoxifen drug-drug interactions that influence CYP450 enzymes [27]. However, this interaction is rarely considered by many traditional or alternative medical researchers. The traditional Chinese BZ formula can mitigate the symptoms of depression, anxiety, and stress-related illness in patients [13]. Recently, mangiferin, a major active component of BZ formula, was found to inhibit CYP450 enzymes in human hepatocytes [17]. Tamoxifen needs to be metabolized into endoxifen, which is considered an active ingredient with antitumor properties, through a pathway mediated by CYP450 enzymes in the liver. This attracted our attention. In the present study, the drug-drug interaction between tamoxifen and BZ formula was explored. We found BZ formula attenuated the efficacy of tamoxifen in breast cancer by modulating CYP450 signaling.

Sufficient research and clinical literature has thoroughly verified that tamoxifen reduces the annual recurrence rate of and reduces the mortality from cancer by approximately 1/2 and 1/3, respectively, in women with ER^+^ breast cancer [28]. For this reason, the Clinical Pharmacogenetics Implementation Consortium recommended tamoxifen as the standard therapy for patients with ER^+^ breast cancer [1]. We found tamoxifen significantly inhibited tumor growth after 3 weeks of treatment in a mouse xenograft model (Fig. 2c). Additionally, tumor growth inhibition by tamoxifen was more than 50% higher than the vehicle group (Fig. 2d-f). However, this remarkable antitumor effect of tamoxifen was significantly inhibited by paroxetine (Fig. 2). These data confirm paroxetine should not be taken during treatment with tamoxifen for ER^+^ breast cancer [9].

Preclinical and clinical data show BZ formula mitigates the symptoms of depression, anxiety, and stress-related illness in patients [13]. The main components of BZ formula are flavonoids, saponins, and phenolic glycosides, such as mangiferin, neomangiferin, isomangiferin, timosaponin BII, timosaponin BII, timosaponin AIII, regaloside A, and regaloside B [15, 16]. Mangiferin has been shown to significantly inhibit CYP450 enzymes in human hepatocytes [17]. We found high dose of BZ formula remarkably decreased the antitumor effects of tamoxifen in a mouse xenograft model (Fig. 2). More importantly, a low dose of BZ formula significantly inhibited tumor cell proliferation (Fig. 2). These data indicated that the antagonistic effect of BZ formula was a dose-dependent. Conversely, we explored the potential influence of tamoxifen on the antidepressant effects of BZ formula and its main components. As an antitussive and sedative traditional Chinese Medicine, *L. brownii* exhibits high inhibitory activity and selectivity toward monoamine oxidase (MAO)-B [29], however, there is no investigation directly confirming its antidepressant effect. And the results shown in Fig. 4a also did not support the antidepressant effects of *L. brownii*. Although more evidence is needed, we believe that the antidepressant effect of BZ formula may originate from *A. asphodeloides* rather than *L. brownii*. However, the synergistic effect of *L. brownii* on the antidepressant effect of *A. asphodeloides* is obvious. We also found mangiferin, timosaponin BII, and timosaponin BIII significantly reduced the duration of immobility in mice (Fig. 4b). The concentrations of the main components of *A. asphodeloides* as determined using HPLC from low to high were timosaponin BII, mangiferin, timosaponin BIII, timosaponin AIII, and then neomangiferin (Fig. 1c and Table 4). Consequently, we analyzed the effect of potential drug-drug interactions between tamoxifen and BZ formula, mangiferin, or timosaponin BII on the antidepressant effect. We found tamoxifen significantly depressed the antidepressant effect of paroxetine and mangiferin, but not BZ formula and timosaponin BII, using the classic tail-suspension test (Fig. 4c). These results confirm BZ formula has a significant antagonistic effect on tamoxifen therapy that may depend on mangiferin, rather than timosaponin B.

Work suggests tamoxifen acts mainly through its active metabolites, endoxifen and 4-hydroxytamoxifen, due to their stronger binding affinity for ERs and better inhibition of cellular proliferation compared to tamoxifen [30]. Our results confirm tamoxifen is mainly metabolized to endoxifen rather than 4-hydroxytamoxifen (Fig. 3a, b). The concentrations of endoxifen and 4-OH-tamoxifen, but not tamoxifen, were obviously lower in the combination groups than the tamoxifen group when normalized to the concentrations of tamoxifen, endoxifen, and 4-OH-tamoxifen in the tamoxifen group after treatment for 4 weeks (Fig. 3). The concentration of 4-OH-tamoxifen increased after treatment for 8 weeks compared to 4 weeks in the tamoxifen group (Fig. 3d). These results indicate that extending the delivery time to maintain or improve drug concentrations in the serum could be beneficial. Nevertheless, the concentrations of endoxifen and 4-OH-tamoxifen were lower in the group receiving tamoxifen and paroxetine or BZ formula compared to the tamoxifen only group (Fig. 3d). These results indicate the disadvantages derived from treating with a combination of tamoxifen and paroxetine or BZ formula increase in the presence of drug antagonism. Additionally, the concentrations of endoxifen and 4-OH-tamoxifen were lower after treatment for 8 weeks compared to 4 weeks of treatment with a combination of tamoxifen and paroxetine (Fig. 3d). This indicates this antagonism is more serious when administration duration is prolonged. The results of the present study indirectly confirm the Food and Drug Administration recommendation of performing CYP2D6 genotyping of ER^+^ patients before tamoxifen treatment in order to prescribe the optimal medication [31]. The present study also supports that these patients should avoid using paroxetine, BZ formula, or other Chinese herbal formulas containing CYP450 inhibitors from *A. asphodeloides* or other sources while receiving tamoxifen.

CYP450 enzymes are the principal enzymes in drug metabolism and play important roles in the metabolism of tamoxifen in the liver. The antitumor effect of tamoxifen depends on its metabolism to endoxifen in the liver [30]. CYP3A4 and CYP2D6 are the predominant metabolic enzymes during this process [3, 4]. Therefore, the potential regulation of CYP450 enzymatic activity by BZ formula and its main active compounds, mangiferin and timosaponin BII, was evaluated using HPLC-MS/MS analysis of rat liver microsomes. We found BZ formula and mangiferin, but not timosaponin BII, significantly inhibited CYP450 activity (Fig. 4b-d). These data further suggest the inhibitory effects on CYP450 activity by BZ formula may derive from flavonoids, rather than saponins. These results indicate that patients should avoid using BZ formula or other Chinese herbal formulas containing *A. asphodeloides* during the use of tamoxifen.

## Conclusions

**Fig. 6:**
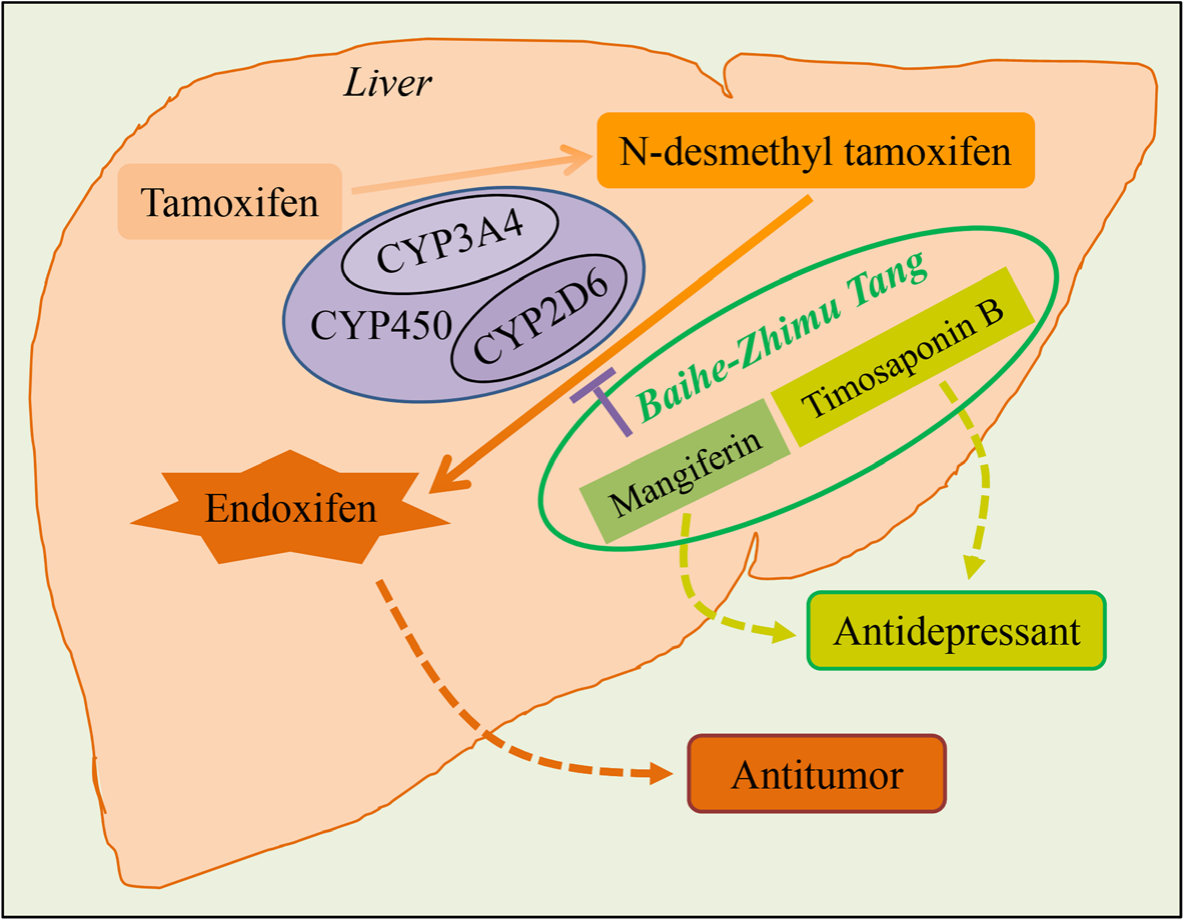
Diagram of the drug-drug interaction between BZ formula and tamoxifen in breast cancer and depression. Tamoxifen is primarily metabolized to N-desmethyltamoxifen in a process mediated by CYP3A4 [3], followed by CYP2D6-mediated oxidation to 4-hydroxy-N-desmethyltamoxifen (endoxifen) [4]. Mangiferin but not timosaponin BII, derived from BZ formula, inhibited P450 activity in rat liver microsomes. BZ formula attenuated the efficacy of tamoxifen against breast cancer. However, tamoxifen did not affect the antidepressant activity of BZ formula or timosaponins, but did inhibit mangiferin. In summary, BZ formula attenuated the efficacy of tamoxifen against breast cancer through CYP450 enzyme activity

BZ formula attenuated the effectiveness of tamoxifen in treatment of breast cancer in mice through the cytochrome P-450 pathway may depend on mangiferin, rather than timosaponin B. While tamoxifen did not affect the antidepressant effect of BZ formula (Fig. 6). The present study laid a foundation for the treatment of patients with breast cancer and depression by BZ formula or other Chinese herbal formulas containing *A. asphodeloides*.

## Data Availability

We declared that materials described in the manuscript, including all relevant raw data, will be freely available to any scientist wishing to use them for non-commercial purposes, without breaching participant confidentiality.

## Abbreviations

4-OH-tamoxifen: 4-hydroxytamoxifen
B: *Lilium brownii var. viridulum* Baker (Baihe in Chinese)
BLI: Bioluminescent imaging
BZ formula: Baihe Zhimu formula or Baihe Zhimu tang
CYP450: Cytochrome P450
ER^+^: Estrogen receptor positive
HPLC: High-performance liquid chromatography
HPLC-MS/MS: High-performance liquid chromatography tandem mass spectrometry
MG: Mangiferin
NMG: Neomangiferin
PX: Paroxetine
TAM: Tamoxifen
TB2: Timosaponin BII
TB3: Timosaponin BIII
Z: *Anemarrhena asphodeloides* Bunge (Zhimu in Chinese)
